# Biochemical profile of *Scenedesmus* isolates, with a main focus on the fatty acid profile

**DOI:** 10.1002/fsn3.4254

**Published:** 2024-05-31

**Authors:** Faezeh Khodadadianzaghmari, Mahshid Jahadi, Mohammad Goli

**Affiliations:** ^1^ Department of Food Science and Technology, Faculty of Agriculture, Isfahan (Khorasgan) Branch Islamic Azad University Isfahan Iran; ^2^ Department of Food Science and Technology, Laser and Biophotonics in Biotechnologies Research Center, Isfahan (Khorasgan) Branch Islamic Azad University Isfahan Iran

**Keywords:** green microalgae, *Scenedesmu*s, α‐Linolenic acid

## Abstract

Biochemical characterization of new microalgal strains that are isolated from diverse environmental conditions is an important starting point for the establishment of high‐quality feedstock for nutraceutical and pharmaceutical applications. In this research study, the biochemical composition of three Iranian native subspecies of *Scenedesmus* microalgae (*Scenedesmus obliquus*, *Scenedesmus bijugusi*, and *Scenedesmus* sp.), with the main focus on fatty acid composition, was studied. The results showed that the strain *Scenedesmus bijugusi* had the highest biomass productivity (48 g/L/d), biomass (0.73%), carbohydrate (13.97%), fat (16.27%), protein (44.04%), chlorophyll‐a (6.32 mg/g), and carotenoids (3.7 mg/g). The lipid profile also revealed that *S. obliquus* had the highest percentage of polyunsaturated fatty acid (46.52%), ratio of ∑n‐3/∑n‐6 (5.96), ratio of polyunsaturated fatty acid to saturated fatty acid (PUFA/SAF) (1.18), α‐linolenic acid (22.74%), hypocholesterolemia index (1.61), and low atherogenic index (0.34). *S. bijugusi* and *S. obliquus*, thus, showed a great promise in nutraceutical and pharmaceutical applications due to their appropriate high productivity, biopigment, protein, lipid, antioxidant activity, long‐chain polyunsaturated fatty acids, and α‐linolenic acid.

## Graphical abstract

The biochemical composition of *Scenedesmus* sp. as a microalga is important due to its satisfactory lipid content for direct use or the use of its metabolites in the food and pharmaceutical industry. *S. bijugusi* and *S. obliquus*, thus, showed a great promise in nutraceutical and pharmaceutical applications due to their appropriate high productivity, biopigment, protein, lipid, antioxidant activity, long‐chain polyunsaturated fatty acids, and α‐linolenic acid.

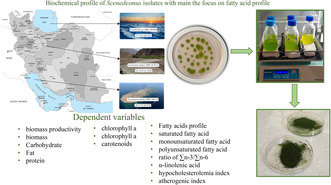

## 1 INTRODUCTION

Microalgae as sources of nutraceuticals and pharmaceuticals are of much interest as they have a high growth rate and do not need fertile agricultural land or freshwater to produce valuable natural metabolites, including fat, antioxidants, fatty acids, pigments, and proteins (Bansemir et al., 2006; Pirastru et al., 2012; Udayan et al., 2021). They are great resources with the potential to revolutionize biotechnology to produce valuable bioproducts for use as nutritive and health components (Lu et al., 2017). The microalga *Scenedesmus* species belongs to the *Scenedesmaceae* family. The genus *Scenedesmus* has about 120 species, and some strains have been successfully used for mass culture and biotechnological applications. It has a rich source of bioactive substrates such as protein, lipid, pigments, and polysaccharide (Banayan et al., 2022; Lu et al., 2017; Sharma et al., 2015; Simioni et al., 2019). The oil content of the most frequently studied *Scenedesmus* varies from 1.9% to 40% (Sharma et al., 2015). In most of the previous research, *Scenedesmus* sp. has been used to produce biofuels and also in aquaculture for the live feeding of fish and larvae and phytoplankton eaters (Patnaik et al., 2019; Udayan et al., 2021). Compared to food and non‐food products, *Scenedesmus* sp. is a very suitable candidate for the commercialization of algal metabolite production (Olia et al., 2022). However, few species of microalgae are used in the industry; further, with high demand and population growth, it is not meeting the requirements, so the local microalgae and biochemical evaluation can be very important (Khosravinia et al., 2023). Exploitation of indigenous strains compatible with the local environment is a logical solution to obtain serious iodine strains with a high potential to produce bioactive compounds from microalga (Dinpazhooh et al., 2022).

Essential fatty acids play a role in coronary heart diseases and strokes; they also play a role in cholesterol metabolism, cancer, blood pressure regulation, and diabetes improvement (Das, 2008). Marine microalgae such as *Scenedesmus* sp., *Chlorella* sp., *Protothecoides*, and *Nannochloropsis Dunaliella salina* have been used for the production of unsaturated fatty acids, such as omega‐3 and α‐linolenic acid (Ötleş & Pire, 2001; Sharma et al., 2015).

Bioprospecting of microalgae is defined as “the identification of economically valuable biochemical resources from algae rich in such content and enables industrial bioresource generation” (Khosravinia et al., 2023). Exploration of native microalgae and evaluation of the competency of bioactive metabolites and their productivity can be, therefore, highly significant. Moreover, native microalgae species of all countries are generally adapted to a wide range of their environments. Therefore, it is important to discover appropriate microalgae species with high biomass and special metabolites like *Dunaliella* sp. for nutraceutical and pharmaceutical applications (Araj‐Shirvani et al., 2024; Gharajeh et al., 2020). Different varieties of isolated microalgae strains were considered for high‐saturated fatty acids for biofuel production (Piligaev et al., 2015) or for pharmaceutical and biotechnological applications (Dinpazhooh et al., 2022).

Owing to the wide variety of natural resources and climate diversity, Iran has been considered as an excellent source for a large number of unstudied microalgal strains with special metabolic abilities. On the other hand, there has been a change of focus among researchers aiming to meet food needs from agriculture to investigate microalgae from different regions, given Iran's climate, vast area of land, and low rainfall. The present research was, therefore, conducted to find out more about the biochemical composition of *Scenedesmus* sp. as a microalga that is important due to its satisfactory lipid content for direct use or the use of its metabolites in the food and pharmaceutical industry. In this research, the biochemical composition of three native subspecies of *Scenedesmus* sp. (*Scenedesmus obliquus*. IBRC‐M‐50130, *Scenedesmus bijugusi*. IBRC‐M‐50116, and *Scenedesmus* sp. IBRC‐M50098), which were isolated from three different regions in Iran (Kharak, Hable Rood River of Garmsar, and Caspian Sea) was investigated. The biochemical compositions of three *Scenedesmus* sp. were described to achieve the main goal of the biotechnological and food industries: producing an economically advantageous nutrient as a substitute for agricultural products in dire situations such as droughts with low environmental damage.

## 2 MATERIALS AND METHODS

### 2.1 Microalgae source

**FIGURE 1:**
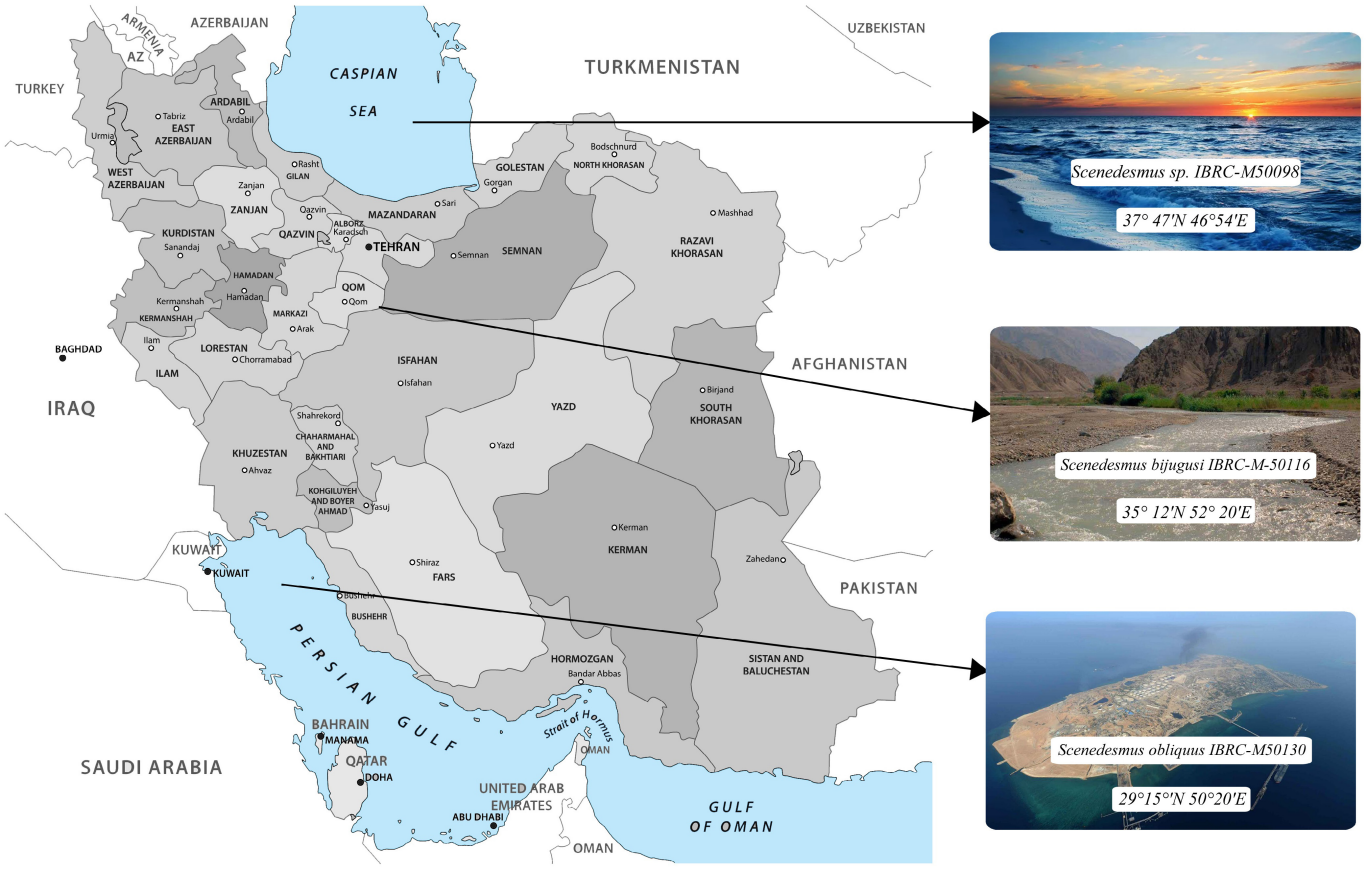
The river map showing the geographical origins of *Scenedesmus obliquus* IBRC‐M50130, from Kharak, 29°15′N 50°20′E, *Scenedesmus bijugusi* IBRC‐M‐50116, Hubbel, River Garmsar, 35°12′N 52°20′E, and *Scenedesmus* sp. IBRC‐M50098 Caspian Sea at 37°47′N 46°54′E.

Three Iranian native *Scenedesmus* isolates, namely, *Scenedesmus obliquus* IBRC‐M50130, from Kharak, 29°15′N 50°20′E, *Scenedesmus bijugusi* IBRC‐M‐50116, Hubbel, River Garmsar, 35°12′N 52°20′E, and *Scenedesmus* sp. IBRC‐M50098 Caspian Sea at 37°47′N 46°54′E, were obtained from the Algal Science Laboratory of Iran Biological Resource Center (Figure 1).

### 2.2 Culture conditions

The isolates were cultured in a Bold Basal's Medium (BBM) containing KH_2_PO_4_ (0.175 g/L), CaCl_2_ 2H_2_O (0.025 g/L), MgSO_4_ (0.075 g/L), K_2_HPO_4_ (0.075 g/L), NaCl (0.025 g/L), H_3_BO_3_ (0.115 g/L), NaNO_3_ (0.25 g/L), Na_2_EDTA (0.001 g/L), KOH (6.2 g/L), FeSO_4_ (4.95 g/L), and H_2_SO_4_ (1 mL/L) at pH 6.8, 10 replicates (Ötleş & Pire, 2001). The cultures were maintained at 25°C, 100 rpm, under a light/dark period of 16/8 h, at 2500 Lux (Kim et al., 2013). After the cultivation period, the biomass was collected, freeze‐dried (Dana Vacuum Industries, Iran), and stored for testing.

### 2.3 Biomass production

The measurement of the dry biomass weight was carried out based on the method proposed by Zhang et al. (2015). The microalgae inoculation (250 mL) was filtered on filter paper (0.45 μm) and then dried in an oven (60°C) for 12 h; finally, it was weighed (Zhang et al., 2015).

### 2.4 Pigment content

Isolated biomass of *Scenedesmus* (100 mg) was added to pure acetone (50 mL) and mixed for 1 h at 50 rpm (Persia‐Med, STM1300, Iran). The samples were centrifuged at 7100 ***g*** for 10 min (Universal 320R, Germany). The upper phase was used to measure chlorophyll a (Ch‐a) (Equation 1), chlorophyll b (Ch‐b) (Equation 2), and carotenoid (Car) (Equation 3) content using spectrophotometry (UV 2100, China) (Alfaia et al., 2021).

(1) $$\mathrm{Ch}-a=11.24A_{662}–2.04A_{645}$$

(2) $$\mathrm{Ch}-b=20.13A_{645}–4.19A_{662}$$

(3) $$\mathrm{Car}=(1000A_{470}-190\mathrm{Ch}-a6314\mathrm{Ch}-b)/214$$

where A_662_, A_645_, and A_470_ are absorbance wavelengths using the spectrophotometer (UV 2100, China) at 662, 645, and 470 nm, respectively. The obtained results were expressed as mg/g.

### 2.5 Total protein

The protein content of *Scenedesmus* sp. was analyzed by the Kjeldahl (Hanan Model 1100, China) method using 5.89 as a conversion factor (AOAC 920.87, 2007; Silva et al., 2021).

### 2.6 Ash content

The dry biomass was ashed in the furnace (PC24 Model, Iran) at 600°C overnight (Liu, 2019).

### 2.7 Carbohydrate content

The measurement of total carbohydrates was done according to Bertrand's method (Heinze & Murneek, 1940).

### 2.8 DPPH radical‐scavenging assay

The ability of the extract to scavenge DPPH (2,2‐diphenyl‐1‐picryl hydroxyl) free radicals was assessed (Silva et al., 2021). Dried powders were extracted with the methanol solution (80%). The extract (0.1 mL) was then mixed with DPPH (0.004%) (4 mL) and incubated at room temperature (30 min). Methanol and DPPH were used as positive controls. Absorbance was measured at 517 nm (UV 2100, China). Radical scavenging activity was expressed as the inhibition percentage (%). Different concentrations of butylated hydroxytoluene (BHT) were then used to draw the standard curve. Total antioxidant was expressed as BHT equivalent (mg/1000 g) (Araj‐Shirvani et al., 2024; Brand‐Williams et al., 1995).

### 2.9 Preparation of the cell extracts for total phenolic

Cells were harvested by centrifugation (Universal 320R, Germany) at 3600 ***g*** for 20 min at 4°C, frozen at −80°C overnight, and freeze‐dried (Dana Vacuum Industries, Iran). The freeze‐dried biomass (0.2 g) was then thoroughly mixed in 5 mL of ethanol/water (3:1 v/v). The tubes were sonicated in an ultrasonic water bath (Parsonic Ultrasonic, Iran) and shaken for 1 h at room temperature. The extracts were centrifuged, and the supernatant was collected (Bulut et al., 2019).

### 2.10 Determination of the total phenolic content

Total phenolic content (TPC) was determined by applying the Folin–Ciocalteuc colorimetric method (Silva et al., 2021). The extracted solution (0.4 mL) was mixed with 2.0 mL of Folin reagent (10%). Then saturated sodium carbonate (2 mL) was added to this mixture. This mixture was obtained after shaking and further centrifuged (Universal 320R, Germany) at 11180 *g* for 10 min; it was allowed to stand for 30 min in the dark and the absorbance was measured at 750 nm by using the UV–vis spectrophotometer (UV 2100, China). The calibration curve (*Y* = 0.0132*x* + 0.0968, *R* ^2^ = .98) of gallic acid was used to calculate TPC. The obtained results were then expressed as gallic acid equivalent (GAE) mg/100 g of the dry weight (DW) of the powder.

### 2.11 Lipid content

Lipid content was extracted by the Soxhlet method and chloroform solvent (CPS6‐Model Iran) (AOAC 920.85, 2007).

### 2.12 Lipid extraction and GC analysis

Fatty acid and lipid analysis was determined by heating the 0.1 g lipid content of the microalgae samples at 80°C using 1 mL of sulfuric acid (2.5%) in methanol (98%) (1:40) for 90 min in screw‐capped tubes. After the addition of 1.5 mL of 0.9% NaCl solution and 0.5 mL of hexane, fatty acids were extracted into the organic phase by shaking; the tubes were centrifuged at a low speed. The samples of the organic phase were separated through gas chromatography (Agilent 6890N Model USA) by applying a flame‐ionization detector (FID) and a temperature program, including an initial temperature of 20°C, which was an increase of 50°C min^−1^ up to 250°C, by using an HP‐88 (Agilent Technologies, USA, 100 m × 0.250 mm × 0.20) column. Helium was used as a carrier gas with a flow rate of 1.2 mL/min and a 1 μL injection volume with a split ratio of 10:1. Each peak was matched with the peak obtained by running the C4:C24 mix (Miquel & Browse, 1992).

### 2.13 Nutritional and health value

Based on the fatty acid composition, the lipid nutritional quality was determined for the isolated *Scenedesmus*. Additionally, the atherogenic index (AI), thrombogenic index (TI), and hypocholesterolemic index (HI) were calculated using the equations ((4), (5), (6)) (Gharajeh et al., 2020).

(4) $$\mathrm{AI}=\left(C12:0+4\times C14:0+C16:0\right)/\left(\sum \text{MUFAs}+\sum \text{PUFAs}\right)$$

(5) $$\mathrm{TI}=\left(C14:0+C16:0+C18:0\right)/(0.5\times \sum \text{MUFAs}+0.5\times \sum n6-\text{PUFAs}+3\times \sum n3-\text{PUFAs}+\left(\sum \text{n3}/\sum \text{n6}\right))$$

(6) $$\begin{array}{c} \mathrm{HI}=(C18:\text{1n9}+C18:\text{2n6}+C18:\text{3n3}+C20:3\\ +C20:\text{4n6}+C20:\text{5n3}+C22:4+C22:\text{6n3})/(C14:0+C16:0) \end{array}$$

where AI is the atherogenic index, TI refers to the thrombogenic index, and HI indicates the hypocholesterolemic index.

### 2.14 Statistical analysis

Statistical comparisons of the samples were performed using the one‐way analysis of variance (ANOVA). Differences between the means were considered significant at the level of 5% (*p* < .05). The data were expressed as means ± standard deviations (SD) of three replicate determinations.

## 3 RESULTS AND DISCUSSION

### 3.1 Biomass dry weight and productivity

The biomass dry weight and productivity of *Scenedesmus obliquus* (0.64 g/L and 42 mg/L/day), *Scenedesmus bijugusi* (0.73 g/L, 48 mg/L/day), and *Scenedesmu*s sp. (0.55 g/L, 36 mg/L/day) are shown in Table 1. The biomass dry weight in our study was approximately similar to that previously recorded for *Scenedesmus* sp. and *Chlorella* sp. (about 1 g/L) (Visca et al., 2017), *Chlorella* (0.015 mg/mL) (Sharma et al., 2015); meanwhile, it was lower than that of *Scenedesmus* spp (4 mg/mL), *Botryococus* (4.5 mg/L) (Sharma et al., 2015), and *Dunalilla* sp. (2.6 mg /L) (Safi et al., 2014). The biomass productivity of *Scenedesmus obliquus* and *Senedesmus abundans* (Piligaev et al., 2015) was also similar to that found in this study. Of course, it should be noted that all 3 *Scenedesmus* isolated in Erlenmeyer grew in non‐aerated and autotrophic conditions.

**TABLE 1:** Biochemical composition (biomass dry weight, protein, carbohydrate, lipid, and ash content) (as % DW)) and biomass productivity (mg/L/d) of isolated *Scenedesmus obliquus* IBRC‐M‐65013, *Scenedesmus bijugusi* IBRC‐M‐50116, *Scenedesmu*s sp. IBRC‐M50098, and other green microalgae.

Isolates	Protein	Carbohydrate	Lipid	Biomass dry weight	Biomass productivity	Ash	References
*Scenedesmus* sp.	1.88^c^ ± 31.42	1.16^c^ ± 9.35	1.45^b^ ± 11.70	0.03^c^ ± 0.55	36	0.59^a^ ± 20.19	This study
*Scenedesmus obliquus*	1.11^b^ ± 39.05	0.51^b^ ± 11.27	1.49^b^ ± 13.20	0.04^b^ ± 0.64	42	1.05^b^ ± 18.17	This study
*Scenedesmus bijugusi*	0.88^a^ ± 44.04	0.87^a^ ± 13.97	0.93^a^ ± 16.27	0.04^a^ ± 0.73	48	0.73^c^ ± 14.89	This study
*Senedesmus obliquus* *Scenedesmus abundans*			41.2 44.4		20.85 73.82		Piligaev et al. (2015)
*Dunaliella salina*	40–57	32	6	–	–	–	Milledge (2011)
*Dunaliella bioculata*	49	4	8	–	–	–	Mäkinen et al. (2017)
*Dunaliella* sp.	34	15	14	–	–	–	Kent et al. (2015)
*Dunaliella* sp.	19–41	4–8	36–47	0.55–1.2	–		Gharajeh et al. (2020)
*Chlamydomonas rheinharrdii*	48	17	21	–	–	–	Christaki et al. (2011)
*Chlorella pyrenoidosa*	57	26	2	–	–	–	Milledge (2011)
*Arthrospira maxima*	60–71	13–16	6–7	–	–	–	Milledge (2011)
*Tetraselmis maculate*	52	15	3	–	–	–	Mäkinen et al. (2017)
*Haematococcus pluvialis*	48	27	15	–	–	–	Bleakley and Hayes (2017)

*Note*: In each column, the averages with different letters are significantly different at the five percent level of the LSD test.

### 3.2 Biochemical composition of the isolates

Protein, carbohydrate, lipid, ash, pigment (chlorophyll‐a, chlorophyll‐b, and carotenoid), total phenol content, and antioxidant activity of the three isolated *Scenedesmus* were measured, as shown in Tables 1 and 2.

**TABLE 2:** Total phenolic, carotenoid, chlorophyll‐a, chlorophyll‐b, total antioxidant, and antioxidant capacity of isolated *Scenedesmus obliquus* (IBRC‐M‐50130), *Scenedesmus bijugusi* (IBRC‐M‐50116), *Scenedesmu*s sp. (IBRC‐M50098), and other green microalgae.

Isolates	Chlorophyll a (mg/g)	Chlorophyll b (mg/g)	Carotenoid (mg/g)	Total phenolic* (mg GAE/g)	Total antioxidant (mg/1000 g)**	Inhibition DPPH %	References
*Scenedesmus* sp.	0.71^b^ ± 4.6	0.62^b^ ± 3.31	0.19 ^b^ ± 1.88	0.06^a^ ± 7.68	37.15 ± 0.57^b^	0.93^b^ ± 60.96	This study
*Scenedesmus obliquus*	0.70^a^ ± 4.55	0.56^a^ ± 4.47	0.76^b^ ± 2.62	1.03^a^ ± 6.59	41.14 ± 3.7^a^	5.9^a^ ± 68.44	This study
*Scenedesmus bijugusi*	0.83^a^ ± 6.32	0.36^c^ ± 1.51	0.21^a^ ± 3.7	0.44^a^ ± 7.09	33.92 ± 1.1^c^	1.80^b^ ± 54.55	This study
*Dunaliella* sp.	10.4	4.7	3.5	–	–	–	Gharajeh et al. (2020)
*Scenedesmus* sp.	–	–	0.15–0.8	5.40	–	25.65	Bulut et al. (2019)
*Dunaliella* sp.	1.17–1.51	2.01–2.5	6.08–7.4	1.68–2.42	26.91–34.54	40.92–55.63	Araj‐Shirvani et al. (2024)

*Note*: In each column, the averages with different letters are significantly different at the five percent level of the LSD test.

* As gallic acid equivalent.

** As BHT equivalent.

#### 3.2.1 Protein content

Proteins are the components of macronutrients essential for growth. Microalgae proteins have a promising future for healthy foods, aquaculture feed, medicine, and pharmaceuticals (Gharajeh et al., 2020). The protein content in *S. bijugusi* (44.05%) was significantly higher than that in *S. obliquus* (39.05%) and *Scenedesmus* sp. (31.42%). As can be seen in Table 1, there is remarkable variability in protein content within green microalgae. The reported protein content for *Senedesmus* species varied from 33.9% in *S. obliquus* (BR003), which was cultivated in a pond, to 65.1% in *S. obliquus* (Amorim et al., 2020) and 53.77% in *Scenedesmus* sp. (de Souza et al., 2020). Meanwhile, the protein content of the isolates *Scenedesmus* (*Scenedesmus obliquus*, *Scenedesmus bijugusi*, and *Scenedesmu*s sp.) was higher than that in animal or vegetable sources such as beef (17%–22%), peanuts (26%), and chicken and fish (19%–24%) (Koyande et al., 2019). The amount of protein production of the three isolated *Senedesmus*, in this research, was also similar to that of *C. vulgaris* (42%–58%) (Safi et al., 2014), while it was lower than that in *Chlamydomonas* (48%), *Chlorell*a (50%–60%), and *Spirulina* (60%–70%) (Gharajeh et al., 2020). However, these results indicated that *Scenedesmus* could be a suitable option for protein production among microalgae, and *S. bijugusi* showed more protein content.

#### 3.2.2 Carbohydrate content

The carbohydrate content of *S. bijugusi* was 13.97%, which was higher than that of *Scenedesmus* sp. (9.35%) and *S. obliguus* (11.27%). The carbohydrate content of the 3 isolates was lower than that of *Chlamydomonas reinhardtii* (17%) (Chen et al., 2013), while it was higher than that of *Dunaliella bioculata* (4%) (Mäkinen et al., 2017) (Table 1). The results, thus, showed that *S. bijugusi* could be a suitable choice for the use of carbohydrates. The carbohydrate of microalgae is, therefore, a good candidate as an alternative to conventional sugars in the food fermentation process (Gharajeh et al., 2020).

#### 3.2.3 Ash content

The amount of ash in *Scenedesmus* sp. (20.2%) was significantly higher than that in the others (Table 1). The lowest amount of ash (14.9%) was observed in *S. bijugusi*. The amount of ash *in Scenedesmus obliguus* was 36.2% (Safi et al., 2014).

#### 3.2.4 Lipid content

The lipid content in *S. bijugusi*, *S. obliguus*, and *Scenedesmus* sp. was 16.27%, 13.2%, and 11.70%, respectively (Table 1). Microalgae fat varies from 2% to 77%, depending on the species and environmental conditions (Safi et al., 2014). The lipid content of the three isolated *Scenedesmus* sp. was similar to that of *Scenedesmus* sp. (16.72%) (de Souza et al., 2020) and *Chlamydomonas rheinharrdii* (21%) (Chen et al., 2013), while it was lower than that of *Dunaliella* sp. (36%–47%) (Gharajeh et al., 2020) and *Scenedesmus obliquus* and *Senedesmus abundans* (Piligaev et al., 2015) (Table 1).

#### 3.2.5 Pigment content

Chlorophyll‐a is the most abundant form of chlorophyll. In photosynthetic organisms, chlorophyll‐a is the primary light‐harvesting complex, in contrast to chlorophyll‐b, which is an auxiliary light‐harvesting pigment (Ünlü et al., 2014). The highest amount of chlorophyll‐a was related to *S. bijugusi* (6.3 mg/g); meanwhile, the highest amount of chlorophyll‐b belonged to *S. obliguus* (4.5 mg/g) (Table 2). *C. vulgaris* can potentially produce chlorophyll‐a and b at 0.25–9.63 and 0.72–5.77 mg/g, respectively (Safi et al., 2014). Considering the study done by Gharajeh et al. (2020) and Araj‐Shirvani et al. (2024) on isolated *Dunaliella* obtained from Iran, the amount of chlorophyll‐a and chlorophyll‐b in the three isolates of *Scenedesmus* of Iran was relatively suitable compared to *Dunaliella* sp. (Table 2).

Carotenoids are part of the photosynthetic apparatus, mainly in the reaction centers of photosystems, where they act as auxiliary pigments for light harvesting processes during photosynthesis and as structural stabilizers for building proteins; these compounds show high antioxidant activity (Swapnil et al., (2021). Carotenoid in *S. bijuguusi* was significantly higher (3.7 mg/g) in comparison to the others (Table 2). Carotenoids content in *Dunaliella* 3.5 mg/g (Gharajeh et al., 2020), 6.08–7.4 mg/g (Araj‐Shirvani et al., 2024) *C. vulgaris* (0.4% DW) (Ru et al., 2020) and *Scenedesmus* sp. (0.15–0.80 mg/g) (Bulut et al., 2019) has been reported. It was observed that the carotenoids content of the three isolates of *Scenedesmus* was appropriate. However, *Scenedesmus* sp. is not a good resource for the extraction of carotenoid (Table 2).

#### 3.2.6 Total phenolic content

Microalgae contain different types of phenolic compounds, such as vanillin, tannic acid, catechin, salicylic acid, ellagic acid, curcumin, quercetin, and benzoic acid. They are a group of bioactive compounds known for their antioxidant, anti‐inflammatory, antimicrobial, anti‐hypertensive, anti‐arthritis, and heart protection activities (Bulut et al., 2019). In this research, no significant difference was observed between the three isolates of *Scenedesmus* sp. (6.5–7.7 mg GAE/g) (Table 2) (*p* > .05). The results obtained in this study were, thus, in good agreement with the phenolic concentration reported by Bulut et al. (2019).

#### 3.2.7 Antioxidant activity

The antioxidant capacity of the three isolates of *Scenedesmus* was evaluated in terms of DPPH radical scavenging capacity. The highest antioxidant activity was recorded for *Scenedesmus obliquus* (68.44%), while the lowest one belonged to *S. bijugusi* (54.55%) (Table 2). The antioxidant activity of *Scenedesmus* sp. 25.65% was reported as well (Bulut et al., 2019), which showed that the 3 isolates of *Scenedesmus* had a more suitable percentage of the DPPH radical scavenging capacity.

### 3.3 Lipid profile of isolates

Sources of marine microalgae are important due to the diversity of the structure and characteristic classification of fatty acids (Kumar et al., 2019). The preliminary fatty acid analysis of all three isolates revealed that the percent of saturated and unsaturated fatty acids in *Scenedesmus* sp. (42.15% and 61%) was significantly different from that of *Scenedesmus bijugusi* (37.37% and 55.42%) and *Scenedesmus obliquus* (39.37% and 52.95%) (*p* < .05) (Table 3). These results were, thus, in line with those of *Scenedesmus* which stated that saturated and unsaturated fatty acids for *Scenedesmus* were 22.34% and 77.66%, respectively (de Souza et al., 2020). The palmitic acid (C16:0), stearic acid (C18:0), and oleic acid (C18:1) contents in *Scenedesmus* sp. (34.14%, 2.43%, and 2.79%) were also significantly different from those of *S. bijugusi*. (22.78%, 0%, and 12.47%) and *S. obliguus* (18.17%, 4.37%, and 6.43%) (*p* < .05) (Table 3). Stearic acid was typically predominant (36.5% and 33.8%) in *Senedesmus abundans* and *Scenedesmus obliquus* (Piligaev et al., 2015). Palmitic acid (C16:0), stearic acid (C18:0), and oleic acid (C18:1) percent in *Scenedesmus* sp. were more than 77% (Tibbetts et al., 2015). The fatty acid profile of *Scenedesmus* sp., *Scenedesmus bijugusi*, and *Scenedesmus obliquus* was close to that of *Scenedesmus* genus, as reported by Tibbetts et al. (2015). Fatty acid isolates of three isolated *Scenedesmus* sp. and *Scenedesmus obliquus* revealed that α‐linolenic acid (C18:3n3) (α‐LA) (25.8%, 22.74%) was dominant in PUFA (Table 4). C18:3n3 (α‐linolenic acid) is an 18‐carbon fatty acid with a double bond in carbons 12, 9, and 15. It is a 3‐n essential fatty acid that is a necessary nutrient for humans and can be obtained through the diet, including α‐linolenic acid, as well as from plant and animal sources. α‐LA can be converted by desaturases and elongates into beneficial 3‐n fatty diacids such as eicosapentaenoic acid (EPA) and docosahexaenoic acid (DHA), which contribute to normal brain development and normal vision. Animals and mammals cannot synthesize α‐LA, while plants are the richest source for providing it (Abedi & Sahari, 2014). Silva et al. (2021) also stated that *S. obliquus* could be regarded as a good source of C18:3n3, C18:2n6, and C18:1n9 (Silva et al., 2021).

**TABLE 3:** Fatty acid profile (%) of isolated *Scenedesmus obliquus* IBRC‐M‐50130, *Scenedesmus bijugusi* IBRC‐M‐50116, *Scenedesmu*s sp. IBRC‐M50098, and other green microalgae.

Isolates	*Scenedesmus* sp.	*Scenedesmus obliquus*	*Scenedesmus bijugusi*	*Spirulina*	*Scenedesmus* sp.	*Tetraselmis* sp.
C16:0	2.12^a^ ± 34.14	0.93^c^ ± 18.17	1.18^b^ ± 22.78	35.34	20.45	24.85
C17:0	0.08^b^ ± 0.25	0.00^b^ ± 0.00	0.34^a^ ± 4.75	–	0.59	–
C18;0	0.85^b^ ± 2.43	0.85^a^ ± 4.37	0.00^c^ ± 0.00	1.47	0.77	0.04
C20:0	0.00^c^ ± 0.00	0.94^b^ ± 5.72	0.95^a^ ± 9.83	–	–	–
C22:0	0.32^b^ ± 1.30	0.43^a^ ± 11.09	0.00^c^ ± 0.00	–	0.85	–
∑SFA	2.89^a^ ± 42.15	1.95^ab^ ± 39.37	1.25^b^ ± 37.37	45.51	25.19	26.43
C18:1	0.58^c^ ± 2.79	0.54^b^ ± 6.43	1.16^a^ ± 12.47	4.51	0.87	27.08
∑MUFA	3.93^a^ ± 21.49	0.54^c^ ± 6.43	1.16^b^ ± 12.47	11.08	19.87	36.32
C18:2 n6	0.00^c^ ± 0.00	0.5^b^ ± 6.68	1.63^a^ ± 14.56	16.87	10.32	21.33
C18:2 n3	1.61^a^ ± 13.71	0.27^b^ ± 4.91	2.62^a^ ± 14.80	–	–	–
C18:3n3	1.96^a^ ± 25.81	1.89^a^ ± 22.74	0.99^b^ ± 13.59	–	39.25	–
C20:3	0.00	12.18 ± 1.47^a^	0.00			
∑PUFA	3.46^c^ ± 39.52	46.52 ± 2.11^a^	42.95 ± 4.68^b^	40.28	54.94	37.26
∑n‐3	3.46 ± 39.52	39.83 ± 1.48	3.61 ± 28.39	–	41.62	11.36
∑n‐6	0.00 ± 0.00	0.50 ± 6.68	1.63 ± 14.56	16.87	13.33	25.9
∑n‐3/∑n‐6	–	5.96	1.94	–	3.12	0.43
PUFA/SFA	0.93	1.18	1.15	0.89	2.18	1.41
Reference	This study	This study	This study	Ötleş and Pire (2001)	Custódio et al. (2014)	Custódio et al. (2014)

*Note*: In each row, the averages with different letters are significantly different at the five percent level of the LSD test.

**TABLE 4:** Atherogenic index (AI), thrombogenic index (TI), hypocholesterolemic index (HI), α‐linolenic acids (α‐LA) of the fatty acid profile of isolated *Scenedesmus obliquus* IBRC‐M‐50130, *Scenedesmus bijugusi* IBRC‐M‐50116, *Scenedesmus* sp. IBRC‐M50098, and other green microalgae.

Isolates	TI	AI	HI	α‐LA	∑ PUFA	References
*Scenedesmus* sp.	0.72	0.55	0.75	25.81	39.52	This study
*Scenedesmus obliquus*	0.57	0.34	1.61	22.74	39.84	This study
*Scenedesmus bijugusi*	1.54	0.41	1.23	13.59	42.95	This study
*Dunaliella* sp. *ABRIINW‐G2/1*	0.2	0.39	1.86	–		Gharajeh et al. (2020)
*Scenedesmus* sp.	–	–	–	39.25	54.94	Custódio et al. (2014)
*Chlorella pyrenoidosa*	–	–	–	18.87	35.48	Ötleş and Pire (2001)
*Chlorella vulgaris*	–	–	–	15.79	38.30	Ötleş and Pire (2001)
*Rhodellaviolacea*	0.24	0.57	1.89	–	–	Aussant et al. (2018)
*Rhodellamaculate*	0.27	0.49	2.01	–	–	Aussant et al. (2018)
*Rhodomonas salina*	0.2	0.91	2.01	–	–	Aussant et al. (2018)

The ratio ∑n‐3/∑n‐6 of *S. obliquus* and *S. bijugusi* is 5.96 and 1.94, respectively (Table 3). Consumption of oily foods is important in a healthy human life, provided that ∑n‐3/∑n 6 fatty acids are balanced; this ratio is considered 5:1 by the World Health Organization (Gharajeh et al., 2020; Rubio‐Rodríguez et al., 2010). The increase in fried foods and fast foods disrupts the balance of this ratio, causing a decrease in ∑n‐3 in foods (Shanab et al., 2018). So, to maintain this balance, increasing the consumption of α‐linolenic acid by enriching food products has been suggested (Silva et al., 2021). *Scenedesmus* has a higher PUFA content, especially as a source of α‐linolenic acid, as compared to other species; *Arthrospira* species, *Chlorella*, and *Dunaliella* (Custódio et al., 2014). *Scenedesmus* is also a rich source of unsaturated fatty acids (Ötleş & Pire, 2001), and *S. bijugusi* and *Scenedesmus* sp. are rich sources of α‐linolenic acid (Table 3).

### 3.4 Lipid nutritional and health value

The atherogenic index (AI), thrombogenic index (TI), and hypocholesterolemic index (HI) of 3 isolated *Scenedesmus* were compared with those of food candidate microalgae (*Dunaliella* sp., *Nannochloropsis oculate*, *Nannochloropsis salina*, *Rhodellaviolacea*, *Dixioniella grisea*, *Rhodellamaculate*, *Rhodomonas salina*, *Isochrysis galbana*, and *Leptocylindrusdanicus*), as shown in Table 4. The lowest AI and the highest HI, which could be an indicator of atherogenic health, play a role in reducing cardiovascular diseases. AI and TI are two important indicators of nutritional health that show platelet aggregation ability. Foods with low AI and TI levels (containing less saturated fatty acids) have a greater ability to protect against such diseases. AI in the oil of *Scenedesmus* sp., *S.obliquus*, and *S. bijugusi* was 0.55, 0.34, and 0.41, respectively, which was partly equal to that of *Dunaliella* sp. *ABRIINW‐G2/1* (0.39) (Table 4). TI and HI values in the three isolates of *Scenedesmus* were approximately comparable with those of *Dunaliella* sp. *ABRIINW‐G2/1* (Gharajeh et al., 2020) and *Rhodomonas salina* (Aussant et al., 2018) (Table 4). Therefore, the health indicators such as AI, TI, and HI (0.34, 0.57, and 1.61) obtained from the fatty acid profile of *S. obliquus* proved the high nutritional value of the fats in these species (Table 4).

## 4 CONCLUSION

The determination of biochemical and bioactive components of the three isolated *Senedesmus* (especially *Scenedesmus bijugusi*. IBRC‐M‐50116 and *Scenedesmus obliquus* IBRC‐M‐50130) of Iran supported their potential nutraceutical and pharmaceutical applications due to medium‐ to high‐protein and high‐quality fats rich in PUFA and α‐linolenic acids, appropriate health indices (high HI and low TI and AI), antioxidant activity, phenolic acid, and biopigment (Chlorophyll). However, it should be pointed out that all 3 isolated *Scenedesmus* were grown in Erlenmeyer under non‐aerated and autotrophic conditions. Undoubtedly, biomass dry weight, protein, lipid content, PUFA, α‐linolenic acids, appropriate health indices (high HI and low TI and AI), antioxidant activity, and phenolic acid of the three isolates would be increased by exposure under an optimized culture condition. Optimizing the growth conditions of these species in order to increase the accumulation of biomass and natural bioactive compounds and using them as whole or cracked cells to enrich food or produce new food products, along with technical and economic evaluations, can be recommended for future studies.

## AUTHOR CONTRIBUTIONS

**Faezeh Khodadadianzaghmari:** Conceptualization (equal); data curation (equal); formal analysis (equal); funding acquisition (equal); investigation (equal); methodology (equal); project administration (equal); resources (equal); visualization (equal); writing – original draft (equal); writing – review and editing (equal). **Mahshid Jahadi:** Conceptualization (equal); data curation (equal); formal analysis (equal); funding acquisition (equal); investigation (equal); methodology (equal); project administration (equal); resources (equal); software (equal); supervision (equal); validation (equal); visualization (equal); writing – original draft (equal); writing – review and editing (equal). **Mohammad Goli:** Conceptualization (equal); data curation (equal); formal analysis (equal); methodology (equal); supervision (equal); visualization (equal); writing – original draft (equal); writing – review and editing (equal).

## CONFLICT OF INTEREST STATEMENT

The authors declare that they do not have any conflict of interest.

## ETHICS STATEMENT

This study did not involve any human or animal testing.

## Data Availability

The data that support the findings of this study are available on request from the corresponding author.
